# A Narrative Review of Osteonecrosis of the Jaw: What a Clinician Should Know

**DOI:** 10.7759/cureus.51183

**Published:** 2023-12-27

**Authors:** Swati Sharma, Rama Shankar, B. Sarat Ravi Kiran, Rohit Breh, Shitun Sarangi, Amitabh Kumar Upadhyay

**Affiliations:** 1 Prosthodontics, Tata Main Hospital, Jamshedpur, IND; 2 Oral and Maxillofacial Surgery, Tata Main Hospital, Jamshedpur, IND; 3 Orthodontics, Tata Main Hospital, Jamshedpur, IND; 4 Dentistry, Tata Main Hospital, Jamshedpur, IND; 5 Medical Oncology, Tata Main Hospital, Jamshedpur, IND

**Keywords:** antiresorptive therapy, mronj, bisphosphonate therapy, osteonecrosis of the jaw, medication-related osteonecrosis of the jaw

## Abstract

Medication-related osteonecrosis of the jaw (MRONJ) is an uncommon complication of antiresorptive therapy (ART) in patients receiving higher and more frequent doses of osteoclast inhibitors. The jaws are the most common site, as they have high bone turnover. The oral structures are exposed to various types of stresses, like mastication and dental diseases, which lead to microtrauma and increased bone remodeling. The hallmark feature of MRONJ is the area of exposed, necrotic, nonhealing, asymptomatic bone for more than eight weeks. Objective signs are pain in the jaw and oral cavity, loose teeth, gingival swelling, ulceration, soft tissue infection, and paresthesia in the trigeminal nerve branches' territory. Clinically, the MRONJ has been defined in four stages, from stage 0 to stage 3. Close coordination between the dentist and oncologist is critical for optimal treatment. Conservative management should be preferred over surgical management. There is significant underreporting and misdiagnosis of MRONJ cases in regular clinical practice. There needs to be more awareness among treating physicians about this sporadic complication of bisphosphonate therapy. This narrative review has given a detailed insight into the subject, starting with etiology, pathogenesis, incidence, clinical presentation, workup, staging, and various management strategies. The review article focuses mainly on practical aspects of MRONJ, which every clinician dealing with the disease must know. With a better awareness of this potential complication, healthcare practitioners dealing with at-risk patients can better diagnose, prevent, address, and provide necessary care.

## Introduction and background

Osteonecrosis of the jaw (ONJ), currently known as medication-related osteonecrosis of the jaw (MRONJ), has been described as an uncommon complication of antiresorptive therapy (ART) for cancer and some benign conditions, as well as with drugs inhibiting angiogenesis [1, 2]. Antiresorptive agents like bisphosphonates (BP) are inorganic pyrophosphates that decrease bone turnover and are used to reduce or delay skeleton-related events (SRE) like fractures in patients with metastatic lytic lesions, hypercalcemia of malignancy, multiple myeloma (MM), Paget's disease, and osteoporosis [1, 2]. There is a higher risk for MRONJ in cancer patients receiving frequent and higher doses of osteoclast inhibitors. The risk is comparatively lesser in patients receiving a lower dose of osteoclast inhibitors for diseases like osteoporosis and Paget's disease [2, 3]. We have compiled facts from numerous previous analyses and extracted the paramount information that every clinician dealing with the disease must know. This narrative review discusses the incidence, risk factors, staging, clinical course, prevention strategies, and management of MRONJ and osteoradionecrosis of the jaw (ORNJ). We do not have data regarding the actual incidence of MRONJ since only a few cases in clinical practice are reported.

Osteonecrosis of the jaw has been mentioned with various nomenclatures, like bisphosphonate-related medication-related osteonecrosis of the jaw (BRMONJ), bisphosphonate-related osteonecrosis (BRON), and most recently, MRONJ [4]. A similar condition known as 'phossy jaw,' also known as phosphorus necrosis, has been reported previously, even before the availability of BPs. The phossy jaw was mainly reported in match makers who were exposed to white phosphorus [5]. The American Association of Oral and Maxillofacial Surgeons (AAOMS) identified jaw necrosis as the basis of complications from other drugs, including receptor activator of nuclear factor kappa beta ligand (RANKL) inhibitor denosumab and antiangiogenic agents, and coined the term medication-related osteonecrosis of the jaw [1-3]. The Multinational Association of Supportive Care in Cancer (MASCC), the International Society of Oral Oncology (ISOO), and the American Society of Clinical Oncology (ASCO) also endorsed this terminology (MRONJ) in a joint guideline [6]. A patient can be diagnosed with MRONJ if there is an ongoing or previous history of exposure to antiresorptive drugs alone or with anti-angiogenesis agents or immune modulators. There should be the presence of frank exposed bone, or bone that can be pointed through an intraoral or extraoral fistula in the maxillofacial region, for more than eight weeks after identification by a healthcare professional with no history of radiation exposure to metastatic disease of the jaw [6].

## Review

Etiology and risk factors

Exposure to antiresorptive medications is primarily responsible for MRONJ. Other factors augment the effect of medications on pathogenesis (Table 1) [7-24].

**Table 1:** Etiology and risk factors for MRONJ mTOR: mammalian target of rapamycin, RANKL: receptor activator of nuclear factor kappa beta ligand, HIV: human immunodeficiency virus; MRONJ: medication-related osteonecrosis of the jaw

Drugs [7-15,21]	Other factors		
	Systemic diseases [16-21]	Dental causes [21-24]	Miscellaneous [21]
Bisphosphonates: ibandronate, risedronate, neridronate, pamidronate, tiludronate, zoledronic acid	Uncontrolled diabetes	Local trauma	Radiation therapy
	Systemic lupus erythematosus	Mandibular exostoses	Atmospheric pressure variations
	Blood dyscrasias	Mylohyoid ridge	Chronic inactivity
	Hypertension	Ill-fitting dentures	Hypersensitivity reactions
Anti-RANKL antibody: denosumab	HIV infection	Neurologic damage	Alcohol abuse
Immunotherapy	Hyperlipidemia	Periapical and periodontal surgery	Tobacco use
Anti-angiogenesis: bevacizumab, sorafenib, suntinib	Rheumatoid arthritis	Poor oral hygiene	Smoking
	Hypothyroidism	Dental implants	Malnutrition
Chemotherapy	Storage diseases	Dental extraction	Hemodialysis
Corticosteroids	Vascular disorders	Periodontitis	Advanced age
mTOR inhibitors: sirolimus, temsirolimus, everolimus	Sickle-cell disease	Subgingival curettage	
	Osteoporosis	Infections	

The primary factor to consider while assessing the risk of developing MRONJ is the cumulative exposure of the patient to BP or denosumab, considering both the dose per treatment and the number of doses given from the start of the treatment [7-15]. There is no proven BP exposure cut-off below, which MRONJ has not been reported to date. Denosumab is more effective than zoledronic acid in increasing bone mineral density in postmenopausal women with osteoporosis and in preventing SREs in patients with cancer with bone metastasis. Osteoporosis studies have shown that BPs accumulate in the bone matrix and have a long-lasting effect for up to three years [7-21]. Denosumab has a shorter half-life of about 28 days and temporarily inhibits bone resorption [12]. There is a faster resolution of MRONJ in patients with solid tumors or multiple myeloma taking denosumab compared to zoledronic acid, as seen in phase 3 studies (median 8.0 vs. 8.7 months) [12].

The prevalence of ONJ is variable and has been reported to range from low to high depending on the indication, duration of therapy, and dosage of antiresorptive medications. The indications of therapy, essential aspects of the disease, approved ART, and risk of MRONJ are described in Table 2 [25-33].

**Table 2:** Indications of ART, approved therapies, and MRONJ risk GLOBOCAN: Global Cancer Observatory, IV: intravenous, SC: subcutaneous, PO: per os, SRE: skeletal-related event; ART: antiresorptive therapy; MRONJ: medication-related osteonecrosis of the jaw

-	Osteoporosis [25,26]	Paget disease of the bone [27]	Prevention of SRE in cancer patients [28,29]	Prevention of hormonal therapy/chemotherapy-induced bone loss [30,31]	Hypercalcemia in cancers [32]	Giant cell tumor of the bones [33]
Important aspects	Common in postmenopausal women and patients on chronic steroid therapy	Chronic metabolic bone disease leading to disorganized bone formation	Many bone metastases originate from disseminated breast, lung, and prostate cancer.	Primarily associated with hormonal therapy for the treatment of breast cancer and prostatic cancers. Chemotherapy also leads to bone loss.	Hypercalcemia is a complication that occurs most commonly in patients with advanced cancers like multiple myeloma, squamous cell carcinomas, etc. It can potentially lead to acute renal failure, altered sensorium, dehydration, and arrhythmias.	Benign locally aggressive tumor with the potential of transforming into a high-grade sarcoma
Disease burden	This is the most prevalent bone disease, leading to approximately 200 million osteoporosis cases and nine million fractures.	It is the second most prevalent metabolic bone disorder worldwide, with a 1.5%–8.3% prevalence rate.	5.1% of cancer cases are diagnosed with metastasis to bone.	Breast cancer is most common at 12.5%, and prostate cancer is fourth most common with 7.8% of all cancer cases in 2020, as per GLOBOCAN 2020 data.	This is reported in about 20%-30% of cancer cases, common in multiple myelomas and squamous cell carcinomas.	It is a rare disease, with 0.1 to one per one million people per year, which affects primarily young adults.
Approved agents and doses	Alendronate 70 mg PO weekly or 10 mg PO once daily; Ibandronate 150 mg PO once a month or 3 mg IV once in 3 months; Risedronate 5 mg PO once a day or 35 mg PO once weekly; Zoledronic acid 5 mg IV once a year; Denosumab 60 mg SC once every six months	Pamidronate 30 mg IV once a day for three consecutive days or 60 mg IV every three months; Risedronate 30 mg PO once daily for two months; Zoledronic acid single dose 5 mg IV	Ibandronate 6 mg IV once every three to four weeks or 50 mg PO daily; Pamidronate 90 mg every four weeks; Zoledronic acid 4 mg IV once every three to four weeks; Denosumab 120 mg SC once every four weeks	Denosumab 60 mg SC once every six months	Ibandronate single IV dose of 2-4 mg; Pamidronate 15-90 mg as a single dose or over 2-4 infusions; Zoledronic acid single dose of 4 mg IV	Denosumab 120 mg SC every four weeks, with an additional 120 mg on days eight and 15 in the first month.
Risk of MRONJ	Low	Low	High	Low	Low	High

The MRONJ incidence depends on the dosage and duration of ART in various indications. Osteoporosis patients receive fewer frequent doses of ART compared to patients with oncological indications where the dosing frequency is high. Therefore, the incidence of MRONJ in osteoporosis is low (0.001% to 0.01%) [7-9] compared to a much higher incidence in oncological indications (0.5% to 4.6%) [10-15]. Overall, greater than 90% of MRONJ cases are reported in cancer patients, which is expected given 12-25 times the dosage of ART compared to osteoporosis [10-15]. Studies have also shown the gradually increasing incidence of MRONJ in cancer patients receiving ART over time. It ranges from 0.5% to 1.1% in year one, 1.2% to 3.7% in year two, and 1.4% to 4.6% in year three [10-15] (Table 3).

**Table 3:** Risk of MRONJ with ART BP: bisphosphonates; MRONJ: medication-related osteonecrosis of the jaw; ART: antiresorptive therapy

Therapy	Incidence
Placebo [8,9]	0-2/10,000
Low-dose oral BP for less than four years [7,8]	10/10,000
Low-dose oral BP for more than four years [7,8]	21/10,000
Low-dose zoledronic acid for three years [7,8]	1.7/10,000
Low-dose denosumab [9]	4/10,000
High-dose zoledronic acid [10,11,13,14]	33-110/10,000
Bevacizumab [15]	20/10,000
High-dose zoledronaic acid + bevacizumab [15]	90/10,000
High-dose denosumab [10,12]	70-190/10,000

Pathophysiology and microbiology

The underlying pathophysiological mechanism behind jaw osteonecrosis has not yet been fully elucidated to date. There are multiple hypotheses, but none have been able to explain all the presented cases of ONJ thoroughly. The use of BP has been hypothesized to cause an excessive reduction in bone turnover due to prolonged inhibition of bone resorption with over-suppression of bone remodeling and associated infection, which leads to bone cell necrosis and apoptosis [34]. In addition, trauma due to the constant mechanical effects of mastication makes the jaw susceptible to the bone turnover suppression effect of BP. Another hypothesis says that MRONJ is vascular apoptosis [34].

Several features make the oral cavity a unique environment. A thin layer of periosteum covers the alveolar bone in both the mandible and the maxilla with an attenuated layer of connective tissues. The jaw is an area of high bone turnover. The alveolar bone has a turnover rate of 10 times that of long bones. Because of the presence of teeth and daily remodeling around the periodontal ligament, the oral structures are subjected to various stresses. These are physiologic in the form of chewing, iatrogenic in the form of various dental procedures, or inflammatory in the form of periodontal diseases in nature. These factors lead to mucosal trauma, bony exposure, and increased bone remodeling [34]. In bone remodeling and turnover, osteoclasts help in the resorption of old bones, and osteoblasts help to form new bones. Bisphosphonates bind to the osteoclast and accumulate at sites of high bone turnover, leading to a higher concentration of bisphosphonates [34].

Furthermore, a complex pathogenic microbial flora colonizes the oral cavity and teeth. Microtrauma during mastication leads to a possible entry portal for pathogenic microbial flora and other inflammatory products into the underlying bone [34, 35]. It is still debatable whether ONJ affects the bone and then affects the mucosa, or vice versa. There is no proven role for any specific microbes in the development of ONJ. Oral flora such as *Actinomyces israelii* is often reported in biopsy specimens of ONJ [35]. Some of these lesions respond to antibiotic therapy; whether the organisms are causative or incidental is unclear.

*Actinomyces *have been reported in histological examinations of necrotic bones in various malignancies by Lugassy et al. and Melo & Obeid [36, 37]. Hansen et al. showed the presence of *Actinomyces *colonies in the histological examination of 43 out of 45 (93.5%) patients with actinomycosis [38]. Histologically, the appearance resembles chronic osteomyelitis, as hypervascular fibrous tissue and inflamed infiltrate fill large intertrabecular spaces.

Bisphosphonates can be taken either intravenously (IV) or orally. It is given monthly for one to two years, followed by three to six monthly for cancers [7-15]. The frequency of administration for osteoporosis is six months to a year. The most commonly prescribed agents include zoledronic acid, pamidronate, alendronate, ibandronate, and risedronate. Denosumab is an anti-RANKL monoclonal antibody that is also associated with ONJ. The development of MRONJ may occur during or after treatment with bisphosphonates and denosumab, which are very effective in the treatment of osteoporosis and cancers of the bones, such as multiple myeloma and advanced solid cancers that have metastasized to the bones [7-15]. The causative association of bisphosphonates and ONJ in patients with postmenopausal osteonecrosis is not proven.

Osteonecrosis of the jaw has been reported to manifest six to 60 months after the initiation of bisphosphonate treatment. The incidence of ONJ is higher in the mandible (73%) than in the maxilla (22.5%), though it can also be detected simultaneously in both (4.5%) and can also affect the hard palate [39].

Osteoradionecrosis

Cancers of the head and neck are often treated with radiation therapy, which compromises the blood flow to the jaws and the healing capacity, particularly at a radiation dose of 60 Grays or more. This is called osteoradionecrosis (ORNJ). The clinical features and treatment are almost similar to MRONJ [40].

Assessment of MRONJ risk

The assessment of the risk of MRONJ is very important for patients. Patients should be assessed and assigned a risk depending upon the factors described subsequently (Table 4) [7-15].

**Table 4:** Risk assessment of MRONJ BP: bisphosphonates; MRONJ: medication-related osteonecrosis of the jaw

Duration of therapy with BP/ denosumab	Low-dose therapy	High-dose therapy	Additional risk factor	MRONJ risk
Less than four years	Yes	No	No	Low
More than four years	Yes	No	No	High
Any	No	Yes	No	High
Any	Yes	No	Yes	High

Clinical presentation

The typical hallmark feature of MRONJ is the area of exposed, necrotic, nonhealing bone, which may remain asymptomatic for more than eight weeks or more. Factual signs can evolve before the clinical manifestation of an obvious ONJ. The signs include a hurried difference in mucosal health, nonhealing mucosa, prolonged ache in the jaw and oral cavity, loose teeth, gingival swelling, erythema, ulceration, soft tissue infection, and paresthesia or anesthesia in the domain of the affected trigeminal nerve branch. If the neurovascular bundles become compressed from the surrounding inflammation, symptoms of altered sensorium, numbness, tingling, and a feeling of a heavy jaw may occur. These signs and symptoms may occur spontaneously or following dentoalveolar surgery. Intraoral or extraoral fistulae may develop when the necrotic maxilla and mandible become secondarily infected [1-5, 21].

In some cases, sharp fragments of bone may cause painful tongue sores, and these fragments of bone poking through the gums may be mistaken for broken teeth. Some cases may present with oral pimples or gum boils, which are sinus tracks without exposed bone. In patients with maxillary bone involvement, chronic maxillary sinusitis secondary to osteonecrosis with or without an oroantral fistula may be the clinical presentation [1-5, 21].

Investigations 

Although the imaging findings are nonspecific, they are essential in the investigation, diagnosis, and management of MRONJ. The various imaging modalities used are as follows [41].

X-ray

X-ray findings of MRONJ are variable and nonspecific, so panoramic radiographs, which can better visualize the presence of ONJ, are preferred.

Cone Beam Tomography (CBCT)

A CBCT is more accurate and sensitive compared to plain radiographs to changes in bone mineralization and demonstrates an area of focal sclerosis, sequestrum formation, and thickening of lamina dura and reactive periosteal bone. However, the extent of lesions is poorly differentiated in the early stages, particularly in nonhealing extraction sockets, periapical radiolucency, and the widening of periodontal ligament space.

Magnetic Resonance Imaging (MRI)

In MRI, on T1-weighted sequences, early disease typically shows decreased signal intensity, and on T2-weighted sequences, it shows intermediate or slightly increased signal intensity. It can show increased or decreased signal intensity in later stages of the disease with variable enhancement findings, which can correlate with the clinical and histological stages of the disease process.

Technetium-99m (Tc99m) Bone Scintigraphy

The imaging findings are nonspecific. Areas of decreased uptake may be present in early disease; in later stages, there is increased uptake with possible decreased central uptake. 

Differential diagnosis

The differential diagnosis for MRONJ is maxillary sinusitis, deep dental caries, alveolar osteitis, gingivitis and periodontitis, periapical abscess, sarcoma, chronic sclerosing osteomyelitis, and osteoradionecrosis [19,41]. 

Staging

The clinical staging system has been adapted and is currently being used by AAOMS, which was recently updated in 2022 and was developed by Ruggiero and colleagues. This system helps to identify the conditions, stages, and characteristics and provides appropriate terminology for diagnosis and management. However, the suggested treatment strategies must be more evidence-based and range from stage 0 to stage 3. The staging is described in Table 5 [41].

**Table 5:** Staging of MRONJ MRONJ: medication-related osteonecrosis of the jaw

Stage	Description
0	Stage 0 is not universally accepted. In 2019, the Multinational Association of Supportive Care in Cancer (MASCC), the International Society of Oral Oncology (ISOO), and the American Society of Clinical Oncology (ASCO) advised that stage 0 should be considered only as an indicator of increased MRONJ risk patients, which could prompt a dental specialist for close follow-up [6].
I	There isexposed asymptomatic bone without meaningful adjoining or regional soft-tissue inflammation or infection.
II	There is exposed bone with associated pain, adjacent or regional soft tissue inflammatory swelling, or secondary infection.
III	There is exposed bone associated with pain, adjacent or regional soft tissue inflammatory swelling, or secondary infection, in addition to a pathologic fracture, extraoral or oral-antral fistula, or radiological evidence of osteolysis extending to the inferior border of the mandible or the maxillary sinus floor.

A recent study concluded that the non-exposed variant of ONJ is the same disease as exposed ONJ [42].

Management

The goal of treatment is to reduce the symptoms. Management of jaw osteonecrosis is a multi-tiered process. Mutual coordination between the treating oncologist and dentist is crucial for the optimal therapy of ONJ and the underlying neoplastic condition. Since wound recovery is impaired in cancer patients with ONJ, a non-surgical strategy may involve further bony involvement and the extension of established lesions.

Preventive Measures

These include patient education, good oral hygiene, a routine clinical dental exam every three to four months, a concise visual assessment of the oral cavity at each follow-up visit, and an orthopantomogram to detect possible dental and periodontal ailments. Apart from conservative preventative measures before initiating treatment, any elective dental or surgical procedure that compromises mucosal surfaces or exposure of bone that cannot entirely recover before starting BP therapy should be avoided. For example, if bisphosphonate therapy can be delayed briefly without risk, in that case, teeth with a poor prognosis or needing extraction should be extracted, additional dental surgeries should be finished, and tissues should be allowed to heal entirely before initiating bisphosphonate treatment [43].

In clinical circumstances, by postponing the use of bisphosphonates, the risks or benefits of therapy have yet to be systemically evaluated. Hence, the treating oncologist must decide to defer bisphosphonate treatment in consultation with a dental specialist. Prophylactic antibiotics are not indicated before the initiation of bisphosphonate therapy. Histological analysis and cultures are not required at this stage. However, antibiotic prophylaxis may be necessary for patients who are at high risk, like those with an indwelling central or peripheral venous catheter and those with a history of endocarditis, artificial cardiac valves, and cardiac murmurs. The dentist should thoroughly check the removable dentures for their potential to cause soft-tissue injury, particularly tissue overlying bone, and adjust if required. Endodontic therapy is preferable to extractions to take out coronal amputations, followed by root canal treatment on retained roots to avoid tooth extraction and the potential development of osteonecrosis [43].

Some investigators have proposed that before dentoalveolar surgery, measuring serum C-terminal collagen peptide levels helps to stratify patients' risk of jaw osteonecrosis. C-terminal collagen peptide is a biomarker of bone turnover, and bisphosphonate therapy is known to suppress levels of C-terminal collagen peptides. Levels less than 100 pg/mL are considered high risk, whereas levels greater than 150 pg/mL are considered minimal risk [44].

Conservative Management

Conservative treatment is the basis of care for patients with ONJ and is mainly applied in the earlier stages of ONJ. Management with an antibacterial mouth rinse like 0.12% chlorhexidine or minocycline hydrochloride for periodontal pockets is advised if gums are infected. Neuropathic pain symptoms can be managed with medications that reduce nerve activity, such as clonazepam, gabapentin, and carbamazepine. In addition to oral antibacterial mouth rinse and pain control, intermittent or continuous systemic antibiotics are indicated in cases of periodontal disease, infections, pain, erythema, or extensive bone exposure or necrosis [45].

Prevention of secondary soft-tissue infection and osteomyelitis is crucial, and antibiotics are indicated for the same. Penicillin remains the preferred drug for antibiotic prophylaxis. A combination of penicillin or amoxicillin/clavulanate and clindamycin or metronidazole is helpful in patients with refractory infections. Azithromycin, or one of the quinolone antibiotics, is a rational choice for patients with penicillin allergies. Cultures are crucial in selecting proper antibiotics. Cultures should include a broad spectrum of aerobic and anaerobic bacteria and fungal or viral cultures if facilities are available. The concerns regarding specific microbes guide the culture pattern. If there are refractory infections, a combination of penicillin, amoxicillin/clavulanate, and clindamycin, or metronidazole is helpful. Antibiotics help to some extent in treating ONJ; however, the specific role of bacterial infection in this situation is imprecise, as pain is usually associated with infection [45].

However, some patients with MRONJ present to clinicians with an exposed bone without apparent pain, inflammation, infection, or any additional signs or symptoms. The decision to treat with an antibiotic and the duration of therapy are not specified at this time. It should be decided by an oral maxillofacial surgeon in consultation with the treating oncologist. However, better mucosal disease control and pain relief have been noted with this approach. Special measures should be taken to avoid or reduce damage to tissues overlying bones. Well-fitting dentures can be used if proper care minimizes soft-tissue trauma or irritation. Patients should clean and remove dentures at night. A removable appliance may be used to shield the uncovered bony areas [45].

Besides, a protecting stent may help patients with uncovered bones that induce trauma to adjoining soft tissues and patients in whom the osteonecrotic site is repeatedly traumatized during routine oral function. A thin vinyl vacuumed mouth guard or delicate acrylic stent may also be utilized, provided the appliance does not cause further trauma to the osteonecrotic area and the patient is competent to maintain oral hygiene. Preventing or reducing trauma and irritation is particularly crucial for patients taking antibiotics. All patients should be observed every three months or more often if manifestation persists or deteriorates and to monitor potential tissue damage closely for any indication of surgical intervention [45].

Teriparatide was helpful in ONJ management in several case reports. In a recent analysis of confirmed MRONJ cases, teriparatide therapy was compared to placebo for eight weeks, leading to a significantly higher number of healed lesions by 52 weeks [46]. Teriparatide treatment is contraindicated in patients with active malignancies or a bone marrow or skeletal radiation history.

Hyperbaric Oxygen

Patients with ORNJ may be treated with antiseptic mouth rinses, antibiotics, surgery, and hyperbaric oxygen. The dosages and duration of hyperbaric oxygen therapy depend upon the ONJ stage and the patient's general condition. Hyperbaric oxygen therapy, previously viewed as nonbeneficial, is now effective in clinical studies [47].

Surgical Management

The oral cavity should be examined adequately for any loose pieces of bone to be removed, which may help with healing. The surgical procedure ranges from minute debridement to complete resection, with a possible primary reconstruction using metallic plates or obturators. Whenever possible, a limited surface bony debridement is to be done to reduce or minimize injury to the surrounding or opposing soft tissues. A tension-free closure is warranted after removing bony fragments and has shown better results. Biopsies are to be avoided unless bony metastasis is presumed. Success with surgical intervention is documented to be better in cases with osteoporosis and MM as compared to solid tumors [48-51].

Antibiotics are appropriate during and after dental surgery and should be continued postoperatively for at least 10 days. Adjuvant therapies have also been suggested, although scientific evidence is usually contentious due to the scarcity of concrete data in randomized controlled studies. In severe cases of ONJ where surgery is required, BP therapy can be interrupted by a joint consultation with the oncologist and dental surgeon. The possibility of a possible augmented risk of skeletal-related events with interruption of BP therapy versus a further increase in the extent of ONJ, if BP therapy is continued, is to be discussed. Since BPs are integrated into the mineral matrix of bone, it is unspecified whether discontinuing BPs provides any benefits in managing ONJ [48-51].

The stage-wise management is summarized in Table 6.

**Table 6:** Management strategies as per stage of MRONJ MRONJ: medication-related osteonecrosis of the jaw

Stage	Management
Stage 0	These are patients at risk who require education, good oral hygiene maintenance, self-care, and preventive measures. Since the symptoms are not specific, the objective is to control the symptoms of pain and infections and closely monitor for signs of progression. Clinical and radiographic monitoring is needed, and intervention is not usually required. The patients must be briefed about the potential of bone exposure and necrosis and be competent to identify manifestations.
Stage 1	If gums are infected, management with an antibacterial mouth rinse is advised. An antibiotic is required to treat dental and periodontal disease, along with a regular follow-up.
Stage 2	If there is necrosis and an associated infection, an antibiotic with an antimicrobial mouthwash and treatment of neuropathic pain are recommended. Barring conservative measures, debridement is usually required.
Stage 3	Surgical management and an antibiotic regimen are needed. The surgical procedure ranges from finite debridement to complete resection and possible primary reconstruction.

Patient-specific considerations for dental surgeons

Osteoporotic Patients About to Start Oral Medical Treatment and Osteoporotic Patients Receiving Oral BP

All patients should be educated regarding the importance of oral hygiene and the risk of developing MRONJ. Periodic follow-up with a dental surgeon is required. Patients should be instructed to quickly report to a dentist for a clinical examination if there are any warning signs and symptoms. In patients treated with oral BP for less than four years without risk elements, postponement of surgery is unwarranted, and all dental procedures can be performed. However, a cautious approach is required for this group. In patients treated with oral BP for less than four years with risk factors or for more than four years, a high-risk approach should be followed and described subsequently.

Asymptomatic Cancer Patients Receiving Chemotherapy and ART

A meticulous oral examination with periodic check-ups every four to six months is warranted. Good oral hygiene is to be maintained to avoid any possibility of dental surgery. To look for any exposed bone or fistula and early signs of MRONJ. An orthopantomography should be done at six- to 12-month intervals to look for osteosclerosis or osteolysis, broadened periodontal ligament areas, or furcation involvements. Any elective invasive procedure that involves a bony injury is to be avoided. In cases of nonrestorable teeth, the crown should be removed, and endodontic treatment should be done for the remaining roots.

Teeth with grades 1-2 mobility should be splinted rather than removed. Teeth with mobility grade 3 and/or endodontic-periodontal lesions should be removed with minimum bony damage and under antibiotic coverage. Antibiotic prophylaxis is indicated in cases where surgical intervention is being done. Deficient dentures should be adjusted, rebased, or substituted, and the biological width should be respected for fixed prosthodontics. Dental implant surgeries and other elective dental surgeries should be avoided.

## Conclusions

The oral structures are subjected to repeated microtrauma and increased bone remodeling. Medication-related osteonecrosis of the jaw is a distinctive complication of ART in patients receiving higher and more frequent doses of osteoclast inhibitors. The jaws are the most common site, being an area of high bone turnover. The hallmark component of MRONJ is the area of exposed and necrotic bone that has been exposed for more than eight weeks and is described in four stages. Close coordination between the dentist and oncologist is critical for optimal therapy. Conservative management should be favored over surgical management. This review describes every aspect of MRONJ in detail and focuses mainly on its practical aspects, which every clinician dealing with the disease must know. With a better awareness of this potential complication, healthcare practitioners dealing with at-risk patients can diagnose, avert, address, and deliver essential care competently.
